# Treatment Outcome in Chronic Hepatitis C Infection: A Four Years Survey Among Iranian Patients

**DOI:** 10.5539/gjhs.v7n3p75

**Published:** 2014-11-30

**Authors:** Aliakbar Hajiaghamohammadi, Rasoul Samimi, Arash Miroliaee, Amir Mohammad Kazemifar, Masoumeh Nazem

**Affiliations:** 1 School of Medicine, Metabolic Diseases Research Center, Qazvin University of Medical Sciences, Qazvin, Iran

**Keywords:** chronic hepatitis C, SVR, Peg-interferon, α-2a pegaferon, ribavirin, treatment

## Abstract

**Background:**

Hepatitis C virus (HCV) infection is universal. Side effects of its treatment are observed in many patients. The present study was designed to evaluate treatment outcome and side effects of the treatment in chronic HCV infection.

**Materials and Methods:**

The current study was conducted prospectively on patients with hepatitis C infection. They had been treated with the standard drug regimen, if indicated. They were followed for treatment response, side effects of therapy, and its related factors.

**Findings:**

From ninety one patients, eighty four persons finished their treatment course. They comprised 71 (84.5%) males and 13 (15.5%) females. Their mean age was 41.5±11.90 years (20–69 years). Genotype 3 was the most common virus genotype (51.2%). Sustained virologic response (SVR) was 84.5% for genotype 3 and 47.5% for genotype 1. Decrease in hemoglobin (43%), weakness and fatigue (26%), neutropenia (13%), and thrombocytopenia (13%) were the most common side effects of the treatment. Seven patients can not finish their treatment course, because of the side effects.

**Conclusion:**

Genotype 3, viral load less than 600000, and more than 3- fold rise in AST are associated with higher SVR. Early administration of the added drugs such as erythropoietin and G-CSF to not reduce the drug doses were also influential.

## 1. Introduction

Chronic hepatitis C virus (HCV) infection is one of the most common chronic liver diseases. Its overall incidence was estimated to be 0.3 per 100,000 in 2010 (Wasley, Grytdal, & Gallagher, 2008; Kenny-Walsh, 1999). The diagnosis of chronic HCV infection is usually made with a reactive HCV antibody test and a positive molecular test that detects the presence of HCV-RNA.

Six major genotypes of HCV have been defined (Lau, Davis, & Prescott, 1996). Genotype 1 is the most common genotype in the United States and Europe (60 to 70 percent of isolates). Genotype 3 is the most common genotype in India, the Far East, and Australia. Genotype 4 is the most common genotype in Africa and the Middle East (Dusheiko, Schmilovitz-Weiss, & Brown, 1994; Ghany, Strader, & Thomas, 2009).

Treatment guidelines for chronic HCV infection were published in 2009 by the American Association for the Study of Liver Diseases (AASLD) (Swain, Lai, & Shiffman, 2010). The goal of antiviral therapy in the patients with chronic HCV infection is to eradicate HCV- RNA, which is predicted by attainment of a sustained virologic response (SVR). The SVR is associated with 97 to 100 percent chance of having negative HCV- RNA test during long-term follow-up. It can therefore be considered cure of the HCV infection (Rutter, Hofer, & Beinhardt, 2013; Bruno & Mangia, 2013; Davis, Wong, McHutchison, Manns, Harvey, & Albrecht, 2003; Manns, McHutchison, Gordon, Rustgi, Shiffman, Reindollar, et al., 2001).

The degree of baseline liver disease, IL-28B genotype, HCV genotype, female gender, age less than 40 years, non-African or non-American race, low body weight (>75 kg), the absence of insulin resistance, and the elevated ALT levels (three-fold higher than the upper limit of normal) are considered important determinants of response to the treatment. Despite these caveats, multivariate analyses have identified two major predictors of SVR among all populations studied, i.e. the viral genotype and pretreatment viral load (Hadziyannis, Sette, Morgan, Balan, Diago, Marcellin, et al., 2004; Coppola, Pisaturo, Tonziello, Sagnelli, Sagnelli, & Angelillo, 2012; Soza, Everhart, Ghany, Doo, Heller, Promrat, et al., 2002).

Various side effects are observed in almost 80 percent of patients receiving peg- interferon and ribavirin combination therapy for chronic HCV infection. In the registration trials of peg- interferon α-2a and 2b plus ribavirin, 10% to 14% of the patients had to discontinue therapy due to an adverse event (Hadziyannis et al., 2004; Coppola et al., 2012). The most common adverse events in these trials had been influenza- like symptoms such as fatigue, headache, fever and rigors, which have occurred in more than half of the patients, and psychiatric side effects (depression, irritability, and insomnia), which have occurred in 22% to 31% of the patients.

Laboratory abnormalities are the most common reasons for reduction of the drugs doses. Among these, neutropenia (absolute neutrophils count [ANC] less than 1500/mm^3^) is a frequent laboratory abnormality, occurring in 18% to 20% of the patients in two large phase III clinical trials, where the dose was reduced 50% for ANC of 750/mm^3^ and permanently discontinued for ANC of 500/mm^3^ (Hadziyannis et al., 2004; Coppola et al., 2012). Severe neutropenia, ANC less than 500/mm^3^ occurred in 4% of the subjects. Despite decline in the neutrophils count, serious infections are uncommon and granulocyte colony stimulating factor is rarely necessary, except in patients with advanced cirrhosis (Afdhal, Dieterich, Pockros, Schiff, Shiffman, Sulkowski, et al., 2004).

Anemia was observed in approximately one-third of the patients, reaching a nadir within 6 to 8 weeks. Dose modification for anemia (hemoglobin level less than 10 g/dl) was required in 9% to 15% in the two phase III registration trials. Growth factors, such as erythropoietin have been used to counter the anemia associated with peg-interferon and ribavirin. Although growth factors improve patient sense of well-being and have reduced the requirement for ribavirin dose reduction, their use has not been shown to improve SVR rates (Shiffman, Salvatore, Hubbard, Price, Sterling, Stravitz, et al., 2007; Fried, Shiffman, Reddy, Smith, Marinos, Goncales, et al., 2002).

The present study was designed to evaluate treatment outcome in chronic HCV infection among Iranian patients.

## 2. Materials and Methods

The current cross sectional study was conducted prospectively on the patients with hepatitis C infection who had attend in hepatitis clinic of Qazvin university of medical sciences, Qazvin, Iran, since 2010 to 2014. They were treated with standard drug regimen 6-12 months, if indicated. Pregnant patients or patients less than 18 years old, and any patients with hemolytic anemia, thalasemia, renal failure, heart failure, uncontrolled diabetes, epilepsy, hyperthyroidism, autoimmune disorders, cytopenia, and cirrhosis were excluded from the study.

The studied patients were evaluated by LFT, CBC, thyroid function test, pregnancy test, liver sonography, the virus genotype test, and the viral load. Their demographic and pertinent data were collected, too.

The patients with genotype 2 or 3 infection were treated with Peg- interferon α-2a (pegaferon produced by Pouyesh pharmaceutical co., Iran) 180 µg SQ (in their arm or thigh) once a week, plus ribavirin 800 mg daily in 4 divided doses. The patients with genotype 1 received Peg- interferon α-2a with similar schedule, plus ribavirin 1000 mg (if they had less than 75 kg weight), or 1200 mg (if they had more than 75 kg weight) daily.

The patients were trained for the disease, the treatment course, and side effects of the drugs. The patients with apparent symptoms of anxiety or depression were consulted by a psychiatrist and were included in the study, if he allowed.

The patients were followed during treatment course every 2 weeks for the first 3 months, and every month then. They were evaluated by CBC and thyroid function test in each visit, too.

The viral load was determined 3 months after the treatment for patients with genotype 1 infection. If it was negative or had more than 2 log reduction, the treatment continued for one year. Otherwise, it was discontinued. For patients with genotype 2 or 3 infection, the treatment was continued for 6 months without determination of viral load.

If neutrophils count was less than 1000 per mm^3^, the patients received G-CSF (granulocyte colony stimulating factor) 1-3 times per week (according to the degree of neutropenia). The treatment discontinued, if neutrophils count was less than 500 per mm^3^.

If hemoglobin was less than 10 g/dl, the patients were administered subcutaneous erythropoietin 1-3 times per week (according to the degree of anemia). The treatment discontinued, if hemoglobin level was less than 8 g/dl. Moreover, the dose of interferon was reduced to half dose, if platelets count was less than 50000 per mm^3^.

The study had been approved by local ethical committee of Qazvin University of medical sciences. The patients provided written informed consent for participation to the study.

Treatment response, side effects of therapy, and its related factors were observed during the study.

## 3. Results

Ninety one patients had the criteria for inclusion in the study. But, 84 patients can finish the treatment course and their data was included in the final report of the study.

Seventy one patients (84.5%) were male and thirteen patients (15.5%) were female. Their mean age was 41.5± 11.90 years (20-69 years). Genotype 3A was the most common virus genotype. It was seen in 43 (51.2%) patients. Genotype 1 was the second prevalent genotype (47.6%) included genotype 1 AB in 16 patients, genotype 1A in 15 patients, and genotype 1B in 9 patients. No genotype 4 was seen (Table 1).

**Table 1:** frequency distribution of the genotype of HCV in the studied patients

Genotype	1a	1b	1ab	2a	3a	4
Number	15	9	16	1	43	0
Percent	17.9	10.7	19.0	1.2	51.2	0

In the lab examination of the patients before start of the treatment, mean AST, ALT, WBC, platelets, and hemoglobin level were 57.26 (15-237), 89.81 (16-650), 6190 (200-6500), 176000 (75000-420000), and 14.23 (10-18.50), respectively. The mean viral load was 860000 (2000-24000000).

SVR was seen in 84.5% of the patients with genotype 3 infection and 47.5% of the patients with genotype 1 infection.

Decrease in hemoglobin (43%), weakness and fatigue (26%), neutropenia (13%), and thrombocytopenia (13%) were the most common side effects of the treatment. Seven patients can not finish their treatment course because of the side effects, even after taking the added drugs.

Forty one patients (51%) developed hemoglobin reduction during the treatment course. In 15 patients, the hemoglobin level was less than 10 g/dl for whom erythropoietin was started. They can finish their treatment course, excluding 2 patients.

Twenty four patients (28.5%) suffered from decreased neutrophils to less than 1500 per mm^3^ during the study. Among them, 16 individuals received G-CSF 2-3 times per week due to having neutrophils less than 1000 per mm^3^. They can end their treatment course, except two patients.

Thirteen patients (15.5%) experienced thrombocytopenia during their treatment course. In only two patients who also had pre-cirrhosis, we ought to stop the treatment, because even half dose of interferon couldn’t reverse thrombocytopenia.

Twenty six patients (31%) developed weakness, fatigue, or irritability during the study. Their symptoms were controlled by pre-treatment training, acetaminophen and/or fluoxetine, except one patient. The results were demonstrated in Table 2.

**Table 2:** Frequency distribution of adverse effects of treatment in the studied patients

	HB Drop	Neutropenia	Thrombocytopenia	Weakness, Fatigue & Irritability
Genotype 1	24	13	7	13
Genotype 3	19	11	6	13
Total	43(51%)	24(28.5%)	13(15.5%)	26(31%)
Discontinuation of treatment	2	2	2	1

32 patients with genotype 1 infection had negative PCR test or more than 2 log fall in viral load 3 months after the treatment (EVR). After 12 months, PCR test of 19 patients (47.5%) were negative.

37 patients (86%) with genotype 3 infection had negative PCR test six months after the treatment. For genotype 2 infection, only 2 patients had negative PCR test six months after the treatment.

Six months after the treatment, 35 of 37 patients (81.4%) with genotype 3 infection and 2 patients with genotype 2 infection attained SVR. The results are shown in Table 3.

**Table 3:** treatment outcome according to genotype of HCV in the studied patients

Genotype	Number	EVR	End of Treatment	SVR
1a	15	13(86.6%)	10(66.6)	8(53.3%)
1b	9	7(77.7%)	5(55.5%)	4(44.4%)
1ab	16	12(75%)	9(56.2%)	7(43.7%)
Total 1	40	32(80%)	24(60%)	19(47.5%)
2a	1	-	1(100%)	1(100%)
3a	43	-	37(86%)	35(81.4%)

Total	84	-	63(73.8%)	55(65.5%)

**Chart 1:**
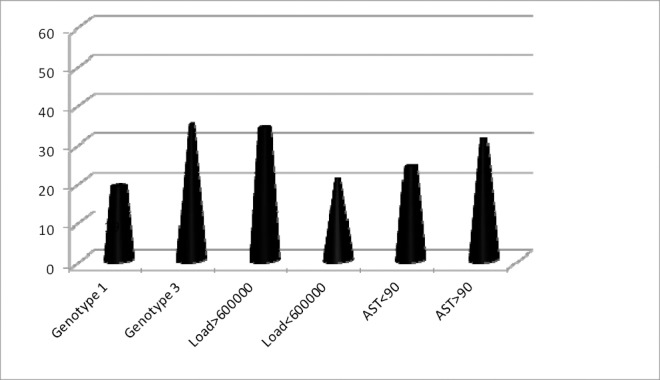
The associated factors of treatment outcome in the studied patients

The statistical analysis evaluated correlation of age and sex of the patients, genotype of HCV, viral load, AST level with the treatment outcome. However, only viral load less than 600000, more than 3-fold rise in AST, and genotype 3 infection were considered as the statistically significant associated factors for the treatment response (p-value less than 0.05). It is demonstrated in Chart 1.

## 4. Discussion

Genotype 1 HCV is the most prevalent genotype in Europe and United State. On the other hand, genotype 3 is the most common genotype in India (Dusheiko et al., 1994; Ghany et al., 2009). Nevertheless, genotype 3 and genotype 1 had the same prevalence in the present study.

Many of our patients had opium use through inhalation route. There was no history of intravenous drug abuse.

In study of Mousavi, Moosavy, Alavian, Eghbali, & Mahboobi in Iran (2013), frequency of HCV genotypes was 316 (62.1%) 1A, 117 (23%) 1B, and 76 (14.9%) 3A. Vossughinia, Goshayeshi, and Rafatpanah (2012) have studied patients with HCV infection in northeast of Iran, they have reported 40% genotype 3A, 39% genotype 1A, and 10.9% genotype 1B among 299 patients.

The most important factor in treatment outcome in HCV infection is sustained virologic response (SVR), due to its impact on the risks of liver-related mortality, hepatocellular carcinoma, and hepatic decompensation. In study of Fried, SVR was higher in non-one genotypes (particularly genotypes 2 and 3), and viral load less than 600000 (16). In study of Tran (2012), SVR was less than 50% for genotype 1. In study of Sarin and Kumar (2012), SVR was more than 70% for genotype 3. In the present study, SVR was 47.5% and 81.4% for genotypes 1 and 3, respectively.

In study of Namazee, Sali, Asadi, Shafiei, Behnava, and Alavian in Iran (2012), half of the patients reached SVR through combined peg-interferon α-2a and ribavirin treatment, the majority of whom had genotype 3A and a minority had genotype 1A. In addition, an age of 40 or lower, non-1A genotype and viral load less than 600 000 IU/ml were strong SVR predictors. In the current study, genotype 3, viral load less than 600 000 IU/ml and more than 3-fold rise in AST were also associated with higher SVR. However, we had not considerable number of patients with genotypes 2 or 4.

Anemia is a common adverse event associated with the use of ribavirin and the recently used HCV protease inhibitors. Ribavirin dose reduction has been recommended in the package insert of pegylated interferon products. On the other hand, the studies have demonstrated the need for maintenance of 80% of the initial ribavirin dose to achieve optimal sustained virologic response (SVR) with dual therapy. The use of erythropoietin-stimulating agents has been shown to be effective for anemia caused by peg- interferon and ribavirin without compromising SVR rates (Hynicka & Heil, 2013). In the current study, anemia was the most common side effect which was occurred in 43% of the patients. Weakness and fatigue, neutropenia, and thrombocytopenia stood in the next orders. Training of the patients about side effects of the drugs and encouraging them to take full dose of the drug during the recommended length and tolerating the side effects will enhance treatment adherence and its success rate. Also, we used the added drugs to combat side effects such as anemia, neutropenia, and thrombocytopenia. In this way, we could continue pegylated interferon and ribavirin with full dose and duration, whenever possible. We discontinued the treatment only in 8.3% of the patients due to the treatment side effects. This figure was 10-14 percent in studies of Manns et al. (2001), and Hadziyannis et al. (2004).

In a systematic review conducted by Alavian, Tabatabaei, and Behnava (2012), data were abstracted from four studies containing 257 patients who developed anemia during therapy. One hundred and twenty six subjects had undergone ribavirin dose reduction. Patients who received erythropoietin in response to hemoglobin drop had a significantly higher probability of achieving SVR compared with those who underwent ribavirin dose reduction because of anemia (relative risk = 1.83 95% CI; 1.41-2.37).

## 5. Conclusion

In summary, the present study suggested that genotype 3, viral load less than 600000, and higher than 3- fold rise in AST are associated with higher SVR. Early administration of the added drugs such as erythropoietin and G-CSF to not reduce the drug doses were also influential to reach a higher rate of SVR.
